# A Comparative Analysis of Bacterial and Fungal Communities in Coastal and Inland Pecan Plantations

**DOI:** 10.3390/microorganisms12071313

**Published:** 2024-06-27

**Authors:** Shijie Zhang, Ting Chen, Yu Chen, Shucheng Li, Wu Wang, Yuqiang Zhao, Cancan Zhu

**Affiliations:** 1 Jiangsu Key Laboratory for the Research and Utilization of Plant Resources, Institute of Botany, Jiangsu Province and Chinese Academy of Sciences (Nanjing Botanical Garden Mem. Sun Yat-Sen), No. 1 Qianhuhoucun, Zhongshanmenwai, Xuanwu District, Nanjing 210014, China; zsjtd310@126.com (S.Z.); ct13347839615@163.com (T.C.); 15150530195@163.com (Y.C.); 2017204015@njau.edu.cn (W.W.); zhaoyuqiang123@126.com (Y.Z.); 2 College of Agriculture, Anhui Science and Technology University, Fengyang, Chuzhou 233100, China; lishucheng12387@126.com

**Keywords:** *Carya illinoinensis*, coastal environments, community diversity and structure, potential functions, soil bacteria and fungi

## Abstract

Pecan forests (*Carya illinoinensis*) are significant contributors to both food and oil production, and thrive in diverse soil environments, including coastal regions. However, the interplay between soil microbes and pecan forest health in coastal environments remains understudied. Therefore, we investigated soil bacterial and fungal diversity in coastal (Dafeng, DF) and inland (Guomei, GM) pecan plantations using high-throughput sequencing. The results revealed a higher microbial diversity in the DF plantation than in the GM plantation, significantly influenced by pH and edaphic factors. The dominant bacterial phyla were Proteobacteria, Acidobacteriota and Bacteroidota in the DF plantation, and Acidobacteriota, Proteobacteria, and Verrucomicrobiota in the GM plantation. *Bacillus*, *Nitrospira* and *UTCFX1* were significantly more abundant bacterial genera in DF soil, whereas *Candidatus Udaeobacter*, *HSB_OF53-F07* and *ADurbBin063-1* were more prevalent in GM soil. Basidiomycota dominated fungal sequences in the GM plantation, with a higher relative abundance of Ascomycota in the DF plantation. Significant differences in fungal genus composition were observed between plantations, with *Scleroderma*, *Hebeloma*, and *Naucoria* being more abundant in DF soil, and *Clavulina*, *Russula*, and *Inocybe* in GM soil. A functional analysis revealed greater carbohydrate metabolism potential in GM plantation bacteria and a higher ectomycorrhizal fungi abundance in DF soil. Significantly positive correlations were detected between certain bacterial and fungal genera and pH and total soluble salt content, suggesting their role in pecan adaptation to coastal environments and saline–alkali stress mitigation. These findings enhance our understanding of soil microbiomes in coastal pecan plantations, and are anticipated to foster ecologically sustainable agroforestry practices and contribute to coastal marshland ecosystem management.

## 1. Introduction

Pecan (*Carya illinoinensis* (Wangenh.) K.Koch: Juglandaceae) is an economically important tree species due to its extensive potential applications, which include seed kernel, oil, wood, and afforestation [1,2]. The indigenous distribution of pecan trees in North America spans from Illinois, USA to Oaxaca, southern Mexico [3,4]. Pecan trees were initially introduced to China in the early 1900s, where they are now predominantly found in the eastern subtropical regions and the Yangtze River Basin, including Yunnan and Jiangsu Provinces [1,5]. Although pecan has recently gained prominence in the Chinese forestry industry, its management faces challenges, such as limited available land for cultivation and the excessive application of chemical fertilizers [1].

Current research on pecans primarily aims to identify optimal management practices [6] through investigating the molecular mechanisms of plant breeding [2], pest and disease management [7], and efficient scion and rootstock grafting [8]. Comparatively little attention is paid to the underground pecan ecosystem [6]. The capacity of a plant to flourish in particular conditions is shaped by its intrinsic genetic makeup, the microbial communities it hosts, and prevailing environmental factors [4]. Soil microbial diversity can be used as an indicator for soil environmental changes and nutrient accumulation [9]. Indeed, bacteria and fungi exhibit dominant regulatory functions in soil nutrient cycling and soil fertility maintenance within forest ecosystems [10]. Recent studies on bacterial and fungal communities in pecan plantations have revealed the effects of pecan age, cultivar selection, soil type, and environmental conditions on microbial community composition [1,6,11].

Due to the rapid growth of the modern economy and society, per capita cultivated land has become increasingly scarce. As a result, preserving existing cultivated land while increasing land output has become a priority, leading to searches for alternative land resources. Despite its saline–alkali soils, coastal land represents a valuable reserve for future development. Currently, attention is focused on the joint application of plants and microbes for the effective revegetation of such land [12]. Many marshlands within coastal ecosystems are treeless due to an elevated soil salinity and alkalinity caused by frequent hydrothermal oscillations, including flooding and drought [13]. Pecans can thrive in diverse soil environments, encompassing acidic and alkaline soils, as well as loamy, moist, rich, sandy, adequately drained, and even heavily clayey soils [14,15]. Pecan trees have recently been proposed as a viable solution for ecological restoration and profitable cultivation in China’s eastern coastal regions. Specifically, pecan cultivation within Jiangsu Province has demonstrated the potential to enhance coastal restoration efforts and deliver significant economic advantages for growers [16]. However, few studies have examined the effects of coastal environments on soil bacterial and fungal diversity in pecan tree soil, and the role of these microorganisms in the adaptability and viability of pecan trees cultivated in coastal environments has not yet been elucidated.

Therefore, it is imperative to compare variation among soil microbial populations between coastal and inland pecan plantations and to analyze the notably enriched microbial communities in coastal regions. Such studies would clarify the potential contribution of dominant microbial species in these coastal areas to improving salt–alkali resistance in host plants. For example, previous studies have identified the increased diversity of rhizosphere microbiome communities as a factor influencing the adaptability of Chinese tallow tree (*Sapium sebiferum*) to coastal environments [13]. Understanding the microbial diversity within pecan plantations is crucial for ensuring tree growth and health, underscoring the pivotal role that microorganisms play in fostering a robust ecosystem. In this study, we employed high-throughput sequencing technology to investigate bacterial and fungal populations in the soil of pecan plantations in Jiangsu Province, China. We hypothesized that soil chemical properties, particularly pH and salt content, would influence bacterial and fungal diversity levels, community composition, and microorganism functions. The objectives of our research were to elucidate the environmental disparities within pecan plantations that influence microbial diversity and community structures, investigate correlations between soil chemical properties and microbial communities, and examine co-occurrence patterns among microbial taxa in relation to coastal and inland environments, alongside exploring potential microbial functions.

## 2. Materials and Methods

### 2.1. Experimental Site and Soil Sample Collection

Soil samples were collected from two pecan plantations located in Jiangsu Province, China. The two sampling sites were about 200 km apart. One sampling site was situated in a pecan plantation on a coastal field within the Dafeng (DF) Forest Farm (33°1′15″ N, 120°46′35″ E) in Yancheng. The other sampling site was in the Guomei (GM) pecan plantation (31°42′22″ N, 119°21′7″ E) in Changzhou. The average age of the pecan trees in each pecan plantation was 6–7 years, and the dominant variety was ‘Pawnee’. In Changzhou, the soil taxonomy is loamy yellow-brown soil, the average annual temperature is 15.8 °C, the annual average precipitation is 1129 mm, the annual average frost-free period is approximately 135 days, and the annual sunshine duration is 1948 h. In Yancheng, the soil taxonomy is sandy soil, influenced by salt and tides, the average annual temperature is 14.4 °C, the annual average precipitation is 1067 mm, the annual average frost-free period is approximately 204 days, and the annual sunshine duration is 2214 h.

We conducted multipoint sampling to ensure a thorough, accurate representation of the soil conditions at each site [17]. Sampling was conducted in early September 2022, at depths ranging from 15 to 30 cm, within a 1 m radius of each pecan tree trunk. To maintain sample independence at each site, the sampled trees were at least 10 m in three different directions from any other pecan tree. The samples were thoroughly mixed, resulting in three mixed-soil samples collected from each pecan plantation. Each mixed-soil sample comprised a combination of 18 soil sampling cores obtained from six trees within the pecan plantation. The soil samples were carefully preserved through storage on dry ice during transportation to the laboratory. Upon arrival, the samples were sieved through a 2 mm mesh to eliminate large debris. The samples were then divided into two portions; one part was stored at −80 °C until soil DNA extraction, and the other was ground and left to dry in a shaded part of the laboratory. This dried portion was subsequently used to analyze soil physicochemical properties.

### 2.2. Determination of Soil Physicochemical Properties

Soil physicochemical properties were mainly determined with reference to agricultural protocols designed in China [18]. These analyses were completed in cooperation with Nanjing Ruiyuan Biotechnology Co., Ltd. (Nanjing, China).

Total nitrogen (TN) was measured using the modified Kjeldahl method. Briefly, a total of 5.0 mL of sulfuric acid (H_2_SO_4_) was added to a soil sample weighing 0.4 g. The mixture was subjected to high-temperature digestion and then analyzed using an automatic Kjeldahl apparatus (SKD-1100; PEIOU Analytical Instruments, Shanghai, China). Available nitrogen (AN) was determined using the alkaline hydrolysis diffusion method. Briefly, the soil was treated with 1.8 mol/L sodium hydroxide solution to convert readily hydrolyzable nitrogen to ammonium nitrogen through alkalolysis; the product was diffused, absorbed with boric acid solution, and titrated with 0.01 mol/L hydrochloric acid standard solution, with methyl red–bromo cresol green used as an indicator. Then, the hydrolyzable nitrogen content was calculated. Total phosphorus (TP) was determined using sodium hydroxide alkali fusion and molybdenum–antimony anti-spectrophotometry [19,20]. Available phosphorus (AP) was extracted from acidic soils using a solution of ammonium fluoride and hydrochloric acid, and from neutral and calcareous soils using sodium bicarbonate solution. The extracted phosphorus was determined through alkali fusion and molybdenum–antimony anti-spectrophotometry. Total potassium (TK) was determined using sodium hydroxide fusion and atomic absorption spectrophotometry [21]. Available potassium (AK) was extracted using a 1.0 mol/L NH_4_OAc solution and subsequently quantified using flame photometry [19].

Soil organic matter (SOM) was determined through oxidation using potassium dichromate (K_2_Cr_2_O_7_) and H_2_SO_4_, followed by titration. The pH was measured using a PHS-3E pH meter (Rex Electric Chemical, Shanghai, China) with a 2.5:1 fresh soil–water ratio. Soil total soluble salts (TSS) were extracted by leaching the soil with water at a water–soil ratio of 5:1. The resulting leachate was evaporated and dried; the obtained weight was taken to represent the total amount of water-soluble salts in the soil. Electrical conductivity (EC) was determined using the electrode method. Briefly, water was added to a sample of naturally dried soil (3.0 g) at a water–soil ratio of 1:5 (*w*/*v*). The mixture was vigorously shaken and allowed to settle, and then the supernatant was carefully filtered through qualitative filter paper. The resulting filtrate was collected, and the EC was measured using a portable conductivity meter (DDBJ-350; Rex Electric Chemical).

### 2.3. DNA Extraction and Polymerase Chain Reaction (PCR) Amplification

Total DNA was extracted from 0.5 g soil samples using the HiPure Soil DNA Kit (Magen, Guangzhou, China) following the manufacturer’s protocols. The integrity of the DNA was assessed through 1% (*wt*/*vol*) agarose gel electrophoresis, and DNA purity and concentration were measured using a NanoDrop 2000 spectrophotometer (Thermo Fisher Scientific, Waltham, MA, USA).

The V3–V4 regions of bacterial 16S rRNA genes and the internal transcribed spacer 2 (ITS2) regions of fungal rRNA genes were amplified using the primers 341F (5′-CCTACGGGNGGCWGCAG-3′) and 806R (5′-GGACTACHVGGGTATCTAAT-3′), and ITS3_KYO2 (5′-GATGAAGAACGYAGYRAA-3′) and ITS4 (5′-TCCTCCGCTTATTGATATGC-3′), respectively [22,23].

PCR was performed as follows: initial denaturation at 95 °C for 5 min, followed by 30 cycles of denaturation at 95 °C for 1 min, annealing at 60 °C for 1 min, and extension at 72 °C for 1 min, with a final extension at 72 °C for 7 min. The PCR procedure used a 50-μL mixture that included 10 μL of 5 × Q5 Reaction Buffer, 10 μL of 5 × Q5 High GC Enhancer, 1.5 μL of 2.5 mM dNTPs, 1.5 μL of each primer (10 μM), 0.2 μL of Q5 High-Fidelity DNA Polymerase, and 50 ng of template DNA. The relevant PCR reagents were sourced from New England Biolabs (Ipswich, MA, USA).

### 2.4. Illumina Sequencing

Amplicons were extracted from 2% agarose gels and purified using the AxyPrep DNA Gel Extraction Kit (Axygen Biosciences, Union City, CA, USA), following the manufacturer’s instructions. Quantification was performed using the ABI StepOnePlus Real-Time PCR System (Life Technologies, Foster City, CA, USA). The purified amplicons were then pooled in equimolar amounts and subjected to paired-end sequencing (PE250) on an Illumina NovaSeq 6000 platform (Illumina, San Diego, CA, USA), according to the standard protocols provided by Guangzhou Gene Denovo Biotech Co., Ltd. (Guangzhou, China).

### 2.5. Sequence Data Processing

The raw sequencing data were processed using the *DADA2* (Divisive Amplicon Denoising Algorithm) package [24] in R v1.14 (R Core Team, Vienna, Austria) to obtain high-quality amplicon sequence variant (ASV) sequences. Briefly, raw reads containing primers or unknown nucleotides (N bases) were filtered and truncated, and a dereplicated list of unique sequences and their abundances, as well as consensus positional quality scores for each unique sequence, were then generated. These consensus scores were used as the basis for the error model used for read denoising by *DADA2*. The paired-end denoised reads were merged to create raw ASVs, ensuring a minimum overlap of 12 bp. Then, the UCHIME algorithm [25] was used to identify and remove chimera sequences. Following chimera removal, the denoised, chimera-free ASV sequences and their abundances were the output. Bioinformatics analysis was conducted using an online platform provided by Guangzhou Gene Denovo Biotech Co., Ltd.

For taxonomic analysis, representative ASV sequences were classified into organisms by a naïve Bayesian model in the RDP Classifier v2.2 software [26], based on the SILVA v138.1 bacterium database [27] and the UNITE v8.3 fungus database [28], and with confidence thresholds ranging from 0.8 to 1.0.

### 2.6. Bioinformatics Analysis

#### 2.6.1. Community Composition Analysis

Abundance statistics for each taxon were visualized using Krona v2.6 [29]. A stacked bar plot was used to represent the community composition results, visualized using the R package *ggplot2* [30]. The species abundance results were plotted as a heatmap using the R package *pheatmap* v1.0.12 [31]. Pearson correlation analysis was conducted using the R package *psych* v1.8.4 [32]. Correlation coefficient networks were generated using the Omicsmart online platform http://www.omicsmart.com (accessed on 11 April 2024).

#### 2.6.2. Indicator Species Analysis

Species were compared between groups using Welch’s *t*-test using the R package *vegan* v2.5.3 [33]. Biomarker features in each group were screened using the R package *lefser* v1.0 [34].

#### 2.6.3. Alpha Diversity Analysis

The Chao1, Shannon, Simpson, and Pielou’s evenness indices were calculated using QIIME v1.9.1 [35]. Alpha indices were compared between groups using Welch’s *t*-test, as implemented in the *vegan* package [33].

#### 2.6.4. Beta Diversity Analysis

Sequence alignment was performed using the R package *muscle* v3.8.31 [36]. Bray–Curtis distance matrix calculation, multivariate statistical analyses, including principal coordinates analysis (PCoA) and non-metric multi-dimensional scaling (NMDS) of Bray–Curtis distances, and Welch’s *t*-test were performed in R using the *vegan* package [33]. Bray–Curtis distances were plotted in R using the *ggplot2* package [30].

#### 2.6.5. Environmental Factor and Network Analysis

To clarify the influence of environmental factors on community composition, canonical correspondence analysis (CCA) was performed using the envfit function in the R package *vegan* [33]. Pearson correlation coefficients between environmental factors and species were calculated using the R package *psych* [32]. A heatmap and correlation coefficient network were generated using Omicsmart.

#### 2.6.6. Kyoto Encyclopedia of Genes and Genomes (KEGG) Functional Analysis

KEGG pathway analysis of the bacterial ASVs was conducted using PICRUSt v2.1.4 [37]. Functional groups, or guilds, of fungi were inferred using FunGuild v1.0 [38]. Functional differences between groups were analyzed using Welch’s *t*-test, as implemented in the R package *vegan* [33].

## 3. Results

### 3.1. Site Characteristics

Significant differences in soil physicochemical properties were observed between the two pecan plantations (Table 1). The levels of AN, TN, AP, AK, and SOM were significantly lower in the DF plantation than in the GM plantation. Conversely, TP, TK, EC, pH, and TSS were significantly higher in DF plantation soil than in GM plantation soil. Salt distribution in the DF plantation soil was not uniform, with a measured salt content of 0.86 g/kg. Despite continuous artificial amelioration efforts aimed at reducing soil salinity, the average salt content and pH (8.04) remained significantly higher in the coastal DF plantation soils than in the inland GM plantation soils, which showed an average salt content of 0.37 g/kg and pH 5.60 (*p* < 0.05).

### 3.2. Soil Bacterial and Fungal Community Diversity and Structure

**Figure 1:**
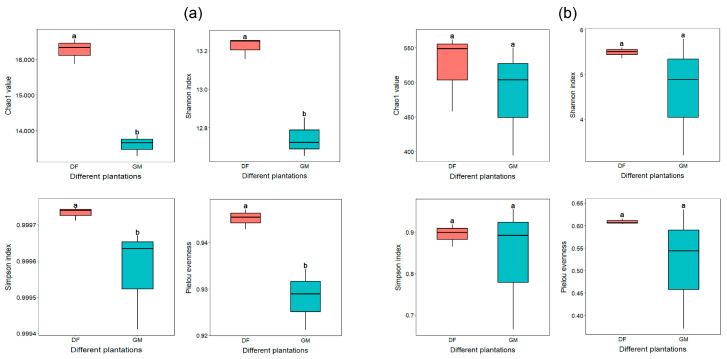
Differences in bacterial (**a**) and fungal (**b**) alpha diversities observed in the soils of two pecan plantations. The height and position of the box plot are determined by the upper and lower quartiles. The median value is represented by the horizontal line inside the box plot, while the maximum and minimum values are indicated by the upper and lower lines outside the box, respectively. Significant differences between the two plantations are denoted by different letters (Welch’s *t*-test, *p* < 0.05). The abbreviations DF and GM correspond to the Dafeng forest farm and the Guomei pecan plantation, respectively.

A total of 524,682 and 672,369 effective tags were identified among the soil bacteria and fungi, respectively, yielding 89,965 bacterial and 3668 fungal ASVs (Table S1). An average of 14,994 bacterial ASVs and 611 fungal ASVs per sample were used to evaluate the richness, diversity, and evenness of soil bacteria and fungi. Based on the Chao1, Shannon, Simpson, and Pielou’s evenness indices, the alpha diversity of bacterial populations in the DF plantation was significantly higher than that in the GM plantation (Figure 1a). However, no significant differences were observed in the alpha diversity of soil fungi between the two plantations (Figure 1b).

**Figure 2:**
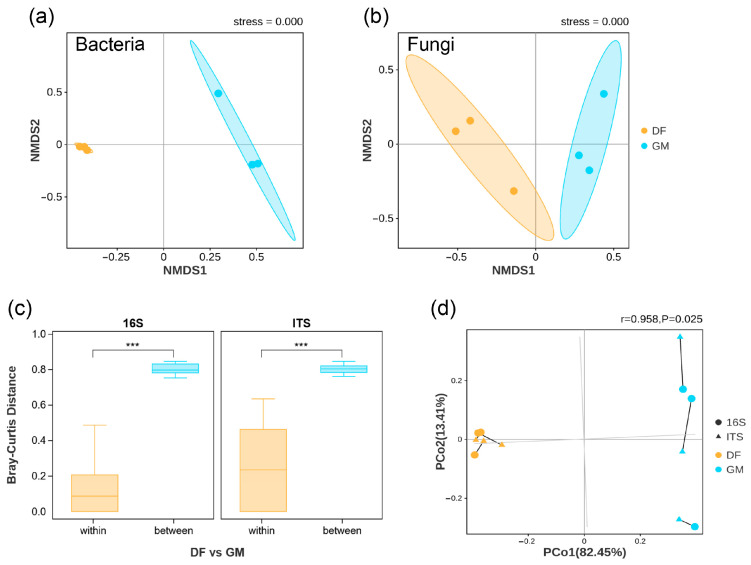
Bacterial and fungal beta diversities in the soils of two pecan plantations were examined. Non-metric multi-dimensional scaling (NMDS) was used to illustrate the microbial community structure for soil bacteria (**a**) and fungi (**b**). Significant differences in beta-diversity of bacteria and fungi were observed between the two plantations. Statistical significance was denoted by “***” (Welch’s *t*-test, *p* < 0.001) (**c**). The Procrustes test was conducted to assess differences in bacterial and fungal responses to the two pecan plantations, based on the results of PCoA (principal co-ordinates analysis) analysis (**d**). The abbreviations DF and GM represent Dafeng forest farm and Guomei pecan plantation, respectively.

The NMDS analysis results clearly demonstrated a distinct separation in the bacterial and fungal community structures between the two plantations (stress = 0.000; Figure 2a,b). Additionally, we assessed microbial beta diversity differences between the plantations on the ASV level using Bray–Curtis distances (Figure 2c). The beta diversity levels of bacteria and fungi showed significant dissimilarity between the two plantations (Welch’s *t*-test, *p* < 0.05). Moreover, our Procrustes analysis indicated that bacterial and fungal responses to these plantation differences were similar (Figure 2d). The two primary PCoA axes, PCo1 (82.45%) and PCo2 (13.41%), accounted for the majority of the variation, indicating that they effectively characterized microbial community composition.

### 3.3. Soil Bacterial and Fungal Community Composition

**Figure 3:**
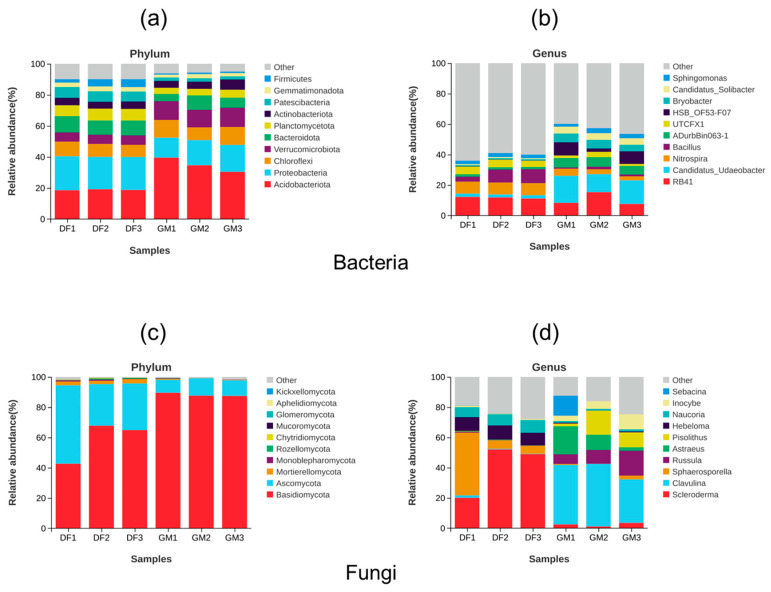
Microbial community composition in the soils of two pecan plantations. Stacked diagram showing the relative abundance of the top 10 bacterial phyla (**a**) and genera (**b**) and the top 10 fungal phyla (**c**) and genera (**d**) identified in different soil samples. The remaining species are uniformly classified under the category of “Other”. The abbreviations DF and GM represent Dafeng forest farm and Guomei pecan plantation, respectively.

The dominant bacterial phyla in the soil of the DF pecan plantation included Proteobacteria (mean: 21.35%), Acidobacteriota (mean: 18.81%), Bacteroidota (mean: 9.71%), and Chloroflexi (mean: 8.53%) (Figure 3a). Among these, Proteobacteria exhibited significantly higher dominance than other phyla (*p* < 0.05) in the soil of the DF pecan plantation (Table S2). In contrast, the predominant bacteria in the soil of the GM pecan plantation consisted primarily of Acidobacteriota (mean: 34.85%), Proteobacteria (mean: 15.48%), Verrucomicrobiota (mean: 15.48%), and Chloroflexi (mean: 10.42%). Acidobacteriota exhibited significantly higher dominance than other phyla (*p* < 0.01) in the soil of the GM pecan plantation (Table S2). At the genus level, *Nitrospira* (mean: 7.85%, *p* < 0.01), *Bacillus* (mean: 6.97%, *p* < 0.05), and *UTCFX1* (mean: 4.76%, *p* < 0.05) had higher relative abundances in DF soil than in GM soil. However, *Candidatus Udaeobacter* (mean: 15.03%, *p* < 0.01), *HSB_OF53-F07* (mean: 6.37%, *p* < 0.05), *ADurbBin063-1* (mean: 5.98%, *p* < 0.001), and *Candidatus Solibacter* (mean: 4.47%, *p* < 0.001) had higher relative abundances in GM soil than in DF soil (Figure 3b and Table S2).

The most dominant phylum among soil fungi was Basidiomycota, which had a significantly higher abundance than other phyla (*p* < 0.05), accounting for 59–88% of all fungal sequences obtained from both plantations (Figure 3c and Table S2). Interestingly, the relative abundance of Ascomycota was significantly higher (*p* < 0.05) in the DF plantation than in the GM plantation, whereas the relative abundance of Basidiomycota was significantly lower (*p* < 0.05) in the DF plantation (Figure 3c and Table S2). Additionally, the DF plantation exhibited a higher relative abundance of Mortierellomycota (Figure 3c). At the genus level, fungal community compositions differed significantly between the two plantations (Figure 3d and Table S2). Specifically, the DF plantation had significantly higher relative abundances of *Scleroderma* (mean: 40.34%, *p* < 0.05), *Hebeloma* (mean: 8.94%, *p* < 0.001), and Naucoria (mean: 7.45%, *p* < 0.001) compared to the GM plantation (Figure 3d and Table S2). In contrast, the GM plantation had significantly higher relative abundances of *Clavulina* (mean: 36.52%, *p* < 0.01), *Russula* (mean: 10.65%, *p* < 0.05) and *Inocybe* (mean: 6.18%, *p* < 0.05) compared to the DF plantation (Figure 3d and Table S2).

### 3.4. Differences in Soil Bacterial and Fungal Community Structure

**Figure 4:**
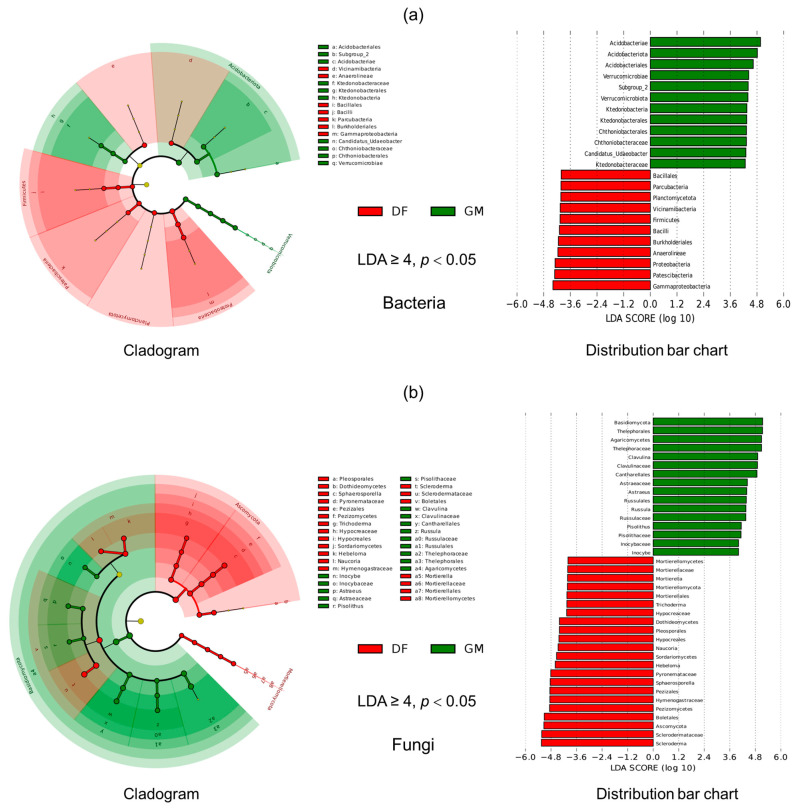
Differences in community composition of soil bacteria (**a**) and fungi (**b**) in two pecan plantations were examined using LEfSe (Linear discriminant analysis Effect Size) analysis. The cladogram on the left illustrates taxonomic levels from kingdom to genus, with each small circle representing a species at that level. The size of the circle is proportional to the species’ relative abundance. Significantly different species (*p* < 0.05) are displayed in red or green, while non-significant species are yellow and their names are not shown. The distribution bar chart in the right panel highlights biomarkers from different groups, with the length of each bar representing the effect size (Linear discriminant analysis (LDA) score) of the differing species. The graph only includes results with an LDA score greater than or equal to 4. The abbreviations DF and GM represent Dafeng forest farm and Guomei pecan plantation, respectively.

The LEfSe results indicated enrichment differences among bacteria and fungi from the phylum to the genus level (Figure 4). In bacterial soil samples, six phyla and one genus exhibited significant enrichment differences according to a linear discriminant analysis (LDA ≥ 4, *p* < 0.05). Similarly, in fungal soil samples, three phyla and 11 genera displayed significant enrichment differences (LDA ≥ 4, *p* < 0.05). Regarding potential bacterial biomarkers, the cladogram illustrates that phylum Proteobacteria exhibited a high level of enrichment in the DF plantation, whereas Acidobacteriota was highly enriched in the GM plantation (Figure 4a). At the genus level, *Ca*. *Udaeobacter* showed significant enrichment in the GM plantation (LDA = 4.3, *p* < 0.05), whereas *Bacillus* was a significant bacterial biomarker in the soil of the DF plantation (LDA = 3.9, *p* < 0.05) (Figure 4a). In contrast to the bacterial findings, fungal biomarkers were more plentiful and showed distinct differences between the two pecan plantations (Figure 4b). *Scleroderma*, *Boletales*, and *Pezizomycetes* were identified as fungal biomarkers in the soil of the DF plantation, whereas *Thelephorales*, *Clavulina*, and *Russula* were fungal biomarkers in the soil of the GM plantation (LDA ≥ 4, *p* < 0.05) (Figure 4b). Notably, the majority of fungal biomarkers from both pecan plantations belonged to the ectomycorrhizal fungal group, despite significantly different species compositions between the two plantations.

### 3.5. Correlation Analysis of Soil Chemical Properties and Microorganisms

**Figure 5:**
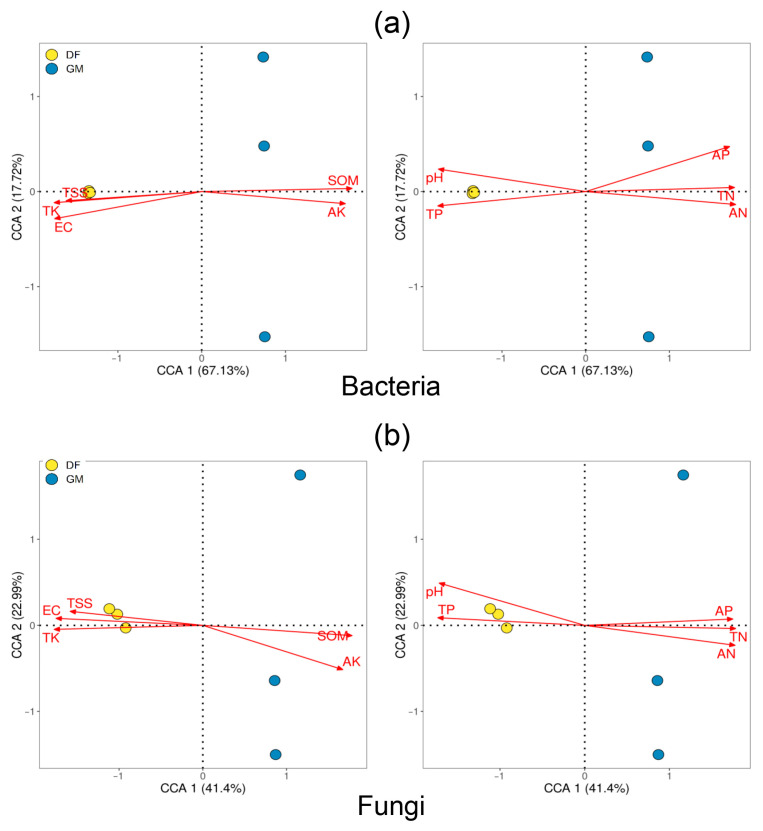
The CCA analysis results of soil bacteria (**a**) and fungi (**b**) and soil chemical characteristics. Different colored and filled circles on the graph represent samples from various pecan plantations, while red arrows represent soil chemical variables. The degree of connection between a soil chemical element and the distribution of communities and microorganisms is indicated by the length of the line between the arrow and the origin. A longer line indicates a higher correlation, and vice versa. The connection between a soil chemical factor and the ranking axes (CCA1 and CCA2, main components 1 and 2) is depicted by the angle between the arrow and the ranking axis. A smaller angle suggests a higher correlation, and vice versa. The abbreviations DF and GM denote Dafeng forest farm and Guomei pecan plantation, respectively.

CCA was conducted to determine the environmental factors responsible for variation in bacterial and fungal communities. CCA yielded two axes, which accounted for 84.86% and 64.39% of the variation in the bacterial and fungal communities, respectively (Figure 5a,b). Bacterial community composition was significantly influenced by the TP, AN, AP, pH, and EC, whereas TN, TK, AK content, and pH were significantly correlated with fungal community composition (*p* < 0.05) (Table S3).

**Figure 6:**
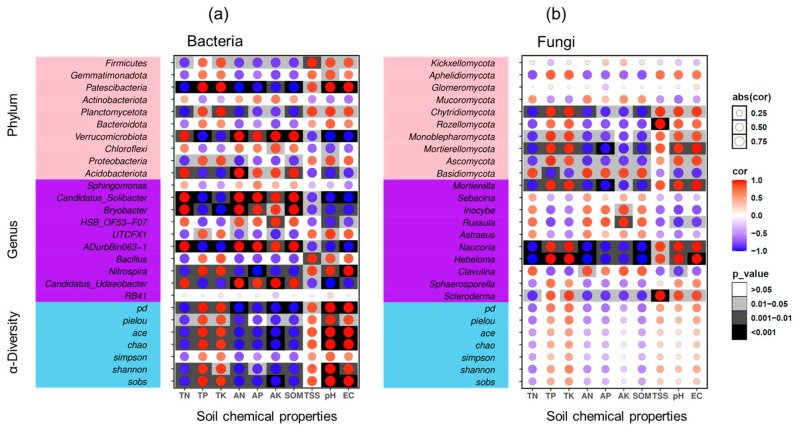
A heatmap illustrating the correlations between soil chemical properties and soil microorganisms’ community is presented. The heatmap focuses on the top 10 genera of dominant bacteria (**a**) and fungi (**b**) (with relative abundances greater than 0.1%). The horizontal and vertical axes represent the soil’s chemical properties, microorganisms and alpha diversity index, respectively. Pearson correlation coefficients are indicated by different colors (cor), with asterisks denoting statistical significance (*p*-value). Abs(cor) is the absolute value of Pearson correlation coefficient. The soil chemical properties include total nitrogen (TN), total phosphorus (TP), total potassium (TK), available nitrogen (AN), available phosphorus (AP), available potassium (AK), soil organic matter (SOM), total soluble salts (TSS), and electrical conductivity (EC). The abbreviations “DF” and “GM” refer to Dafeng forest farm and Guomei pecan plantation, respectively.

The results of Pearson correlation analysis between soil microorganisms and soil chemical properties are presented in Figure 6. The heatmap highlights the 10 genera and phyla, with relative abundances exceeding 0.1%. Generally, TP, TK, TSS content, pH and EC were positively correlated with bacterial alpha diversity indices, whereas TN, AN, AP, AK, and SOM content were negatively correlated with fungal alpha diversity indices.

Within the bacterial community, the dominant phylum in DF plantation soil (Proteobacteria) was significantly positively correlated with soil TP and TK (*p* < 0.05), TSS (*p* < 0.001), pH (*p* < 0.05), and EC. Conversely, the dominant phylum in GM plantation soil (Acidobacteriota) was significantly positively correlated with soil TN (*p* < 0.05), AN (*p* < 0.001), AP (*p* < 0.05), AK (*p* < 0.05), and SOM (*p* < 0.01) content (Figure 6a). Specifically, several microbial genera were significantly correlated with soil properties. *Bacillus* and *Nitrospira* were significantly positively correlated with soil pH (*p* < 0.01), whereas *HSB_OF53-F07* was significantly negatively correlated with soil pH (*p* < 0.05). *Nitrospira* and *UTCFX1* were significantly positively correlated with TSS, whereas *ADurbBin063-1*, *HSB_OF53-F07*, and *Ca. Udaeobacter* were significantly negatively correlated with TSS (*p* < 0.05). *UTCFX1* was significantly positively correlated with TP, whereas *Ca. Solibacter* and *Ca. Udaeobacter* were significantly negatively correlated with TP (*p* < 0.05). *Bryobacter* was significantly negatively correlated with TK, and *Ca*. *Solibacter* was significantly negatively correlated with soil EC (*p* < 0.01). Moreover, *Ca. Solibacter* and *Ca. Udaeobacter* were significantly positively correlated with soil AP (*p* < 0.05), whereas *UTCFX1* was significantly negatively correlated with soil AP (*p* < 0.05). *Bacillus* and *Nitrospira* were significantly negatively correlated with soil AK, whereas *Ca. Udaeobacter* was significantly positively correlated with soil AK (*p* < 0.05). *Bryobacter* was significantly positively correlated with soil TN, AN, and SOM (*p* < 0.05) (Figure 6a).

In the fungal community, Ascomycota and Basidiomycota exhibited significant opposite correlations with soil properties. At the genus level, *Scleroderma*, *Naucoria*, and *Hebeloma* were significantly positively correlated with TSS, whereas *Inocybe* and *Russula* were significantly negatively correlated with TSS (*p* < 0.05). Additionally, *Sphaerosporella*, *Scleroderma*, and *Hebeloma* were significantly positively correlated with soil TK (*p* < 0.05). *Mortierella* was significantly positively correlated with soil EC (*p* < 0.05). *Hebeloma* was significantly positively correlated with soil TP but significantly negatively correlated with soil AP (*p* < 0.05). Moreover, *Sphaerosporella*, *Scleroderma*, and *Hebeloma* were significantly negatively correlated with soil AN, TN, and SOM (*p* < 0.05) (Figure 6b).

### 3.6. Soil Microorganism Correlation Network Structures

**Figure 7:**
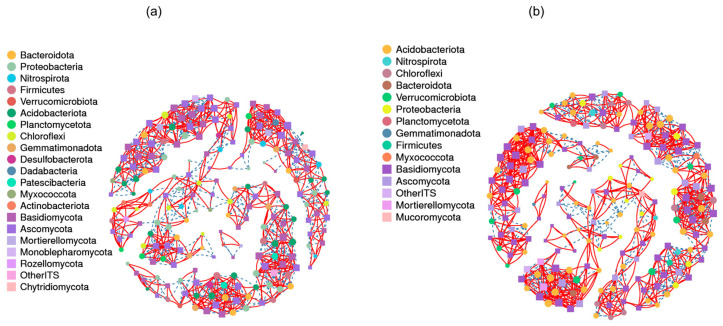
Results of co-occurrence network analysis of dominant bacteria and fungi in DF pecan plantation (**a**) and GM pecan plantation (**b**). Bacterial (Filled circle) and fungal (Filled square) co-occurring networks are shown for each group based on correlation analysis, with the top 500 ASVs selected. Co-occurring networks colored by modularity class. Nodes represent ASVs, and links stand for strong (Pearson’s ρ > 0.9) and significant (*p* < 0.05) correlation. The size of each node is proportional to the degree; solid red links represent positive correlation; blue dotted links represent negative correlation.

A correlation network analysis revealed 396 and 399 total microbial nodes, and 1058 and 1086 total microbial links, in the DF and GM plantations, respectively (Figure 7 and Table S4). A nodal species clustering analysis showed that the DF plantation was primarily characterized by the dominance of Bacteroidota, Proteobacteria, and Nitrospirota (Figure 7a). In contrast, the GM plantation exhibited high prevalence of Acidobacteriota, Nitrospirota, and Chloroflexi (Figure 7b). Within both plantations, Basidiomycota and Ascomycota were identified as the dominant fungal phyla (Figure 7). Regardless of the plantation, the majority of links detected (48.6% and 51.4%) were identified as 16S–ITS links (Figure 7; Table S4), which suggests that 16S–ITS link patterns play a prominent role in shaping the microbial network. In the DF plantation, the proportions of positive (51.8%) and negative (48.2%) links were similar (Table S4). However, there was a significant disparity in the GM plantation, with a notably higher proportion of positive links (64.6%) compared to negative links (35.4%) (Table S4). Overall, the prevalence of positive links exceeded that of negative links, indicating the dominant influence of co-occurrence patterns in the microbial network. Notably, these co-occurrence and mutual exclusion patterns undergo changes in response to environmental factors. Furthermore, soil in the GM plantation exhibited simpler network topologies for the top 500 ASVs of both bacterial and fungal communities compared to those in DF plantations (Table S5).

Our analysis of degree scores identified five keystone taxa at the bacterial genus level in DF plantations: *RB41*, *Bacillus*, *UTCFX1*, unclassified Vicinamibacteraceae, and *Chryseolinea*. In the GM plantation, the keystone taxa were *RB41*, *Bryobacter*, *Ca. Udaeobacter*, *Ca. Xiphinematobacter* and an unclassified Acidobacteriaceae subgroup (Table S6). The most significant fungal genera in the DF plantation were *Scleroderma* (particularly *Scleroderma areolatum*), *Striaticonidium* (particularly *Striaticonidium brachysporum*), *Nigrograna*, *Paulisebacina*, and *Hebeloma*. The key fungal genera in the GM plantation were *Sebacina*, *Mycena*, an unclassified Agaricales genus, and *Mortierella* (Table S6).

### 3.7. Functional Analyses of Soil Bacteria and Fungi

**Figure 8:**
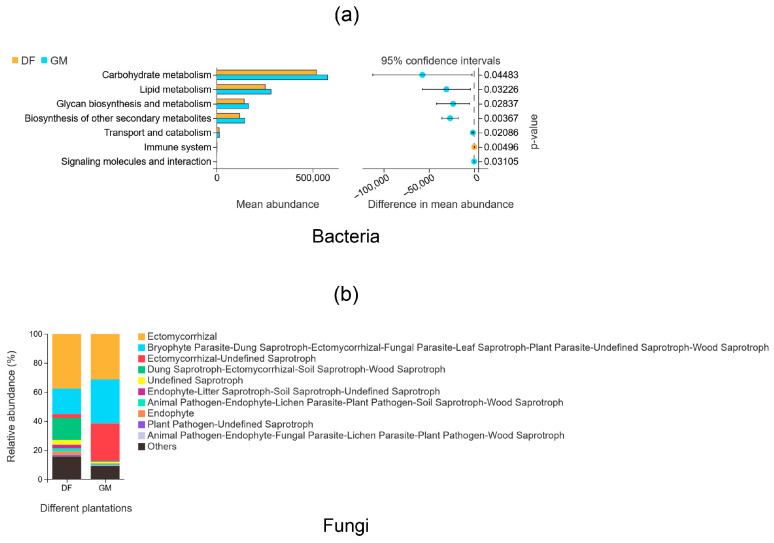
Functional predictions of bacteria and fungi in the soil of two pecan plantations. (**a**) The functional predictions of bacteria were determined using PICRUSt2 analysis. The left half of the graph depicts the differential function, with the ordinate representing its abundance, while the abscissa represents the differential functional abundance. On the right half, the abscissa represents the confidence interval range of the difference in functional abundance between groups. The high-abundance grouping is indicated by color, and the ordinate represents the *p* value (Welch’s *t* test, *p* < 0.05). (**b**) The functional predictions of fungi were determined using FunGuild analysis. The vertical axis represents functional abundance, and the horizontal axis represents groups. The graph shows the top 10 functional abundances, and the remaining functional abundances are uniformly classified under the category of “Other”.

PICRUSt2 and FunGuild were used to predict the functions of bacterial and fungal communities, respectively; the results are shown in Figure 8. In our study, all ASVs were used for conducting functional predictions. Specifically, in the bacterial community, 116,228 ASVs were included in the analysis. A total of 100% of the bacterial ASVs were predicted to have EC annotations and KOs. For the fungal community, ASVs annotated with FunGuild functions represented 48% of all ASVs. The analysis revealed that the bacterial community had the highest functional abundances in carbohydrate metabolism, lipid metabolism, glycan biosynthesis and metabolism, and the biosynthesis of other secondary metabolisms (Figure 8a). Importantly, soil bacteria in the GM plantation had a greater capability for carbohydrate metabolism than those in the DF plantation (Figure 8a). FunGuild analysis indicated that ectomycorrhizal fungi had the highest relative abundance, with the DF plantation showing a higher abundance (approximately 37%) than the GM plantation (approximately 31%) (Figure 8b). However, the relative abundances of two functional groups, one comprising bryophyte parasitism, dung saprotrophism, ectomycorrhizal symbiosis, fungal parasitism, leaf saprotrophism, plant parasitism, and wood saprotrophism and the other comprising ectomycorrhizal symbiosis, and undefined saprotrophism, were higher in GM plantation soil than in DF plantation soil. In contrast, the functional group comprising dung saprotrophism, ectomycorrhizal symbiosis, soil saprotrophism, and wood saprotrophism was more abundant in DF plantation soil than in GM plantation soil (Figure 8b).

## 4. Discussion

### 4.1. Variation in Microbial Diversity between Coastal and Inland Pecan Plantations

Under varying soil conditions, plants attract different microbial communities. Plants and their associated microbes establish a balanced or mutualistic relationship, allowing them to thrive in diverse soil environments [39]. In this study, soil bacterial diversity in the DF plantation was significantly higher than that in the GM plantation (*p* < 0.05); although no significant differences in soil fungi diversity were observed between the two plantations, fungal diversity was slightly higher in the DF plantation than in the GM plantation (Figure 1). These results suggest that the adaptability of pecan trees to a coastal environment may be enhanced by more abundant microbial interactions in this study. Recent studies have also revealed that the soil of *Sapium sebiferum* trees exhibits significantly greater bacterial community diversity in coastal environments than in forestry nursery environments [13].

The beta diversity analyses revealed significant dissimilarity in bacterial and fungal communities between the two plantations in this study (Figure 2). These findings suggest that, despite the apparent similarity in fungal alpha diversity, there are notable differences in the species composition of both fungal and bacterial communities between the two pecan plantations. These compositional differences are likely driven by the distinct soil environmental conditions of the inland and coastal plantations. Therefore, complex tree–microbe interactions have the potential to strengthen tree resistance against inhospitable environments. Notably, trees can enhance their adaptability to coastal environments by selectively recruiting microbial species that display a high tolerance for salinity [40].

### 4.2. Taxonomic Composition and Structural Changes in Soil Microbial Communities of Pecan Plantations

A diverse array of bacteria populate the soil surrounding plant roots and are widely acknowledged for their pivotal role in regulating nutrient cycling [41]. In our study, regardless of pecan plantations, Acidobacteriota and Proteobacteria were identified as the dominant bacterial phyla. This observation aligns with previous findings in different soil types [13,42]. Our results demonstrate the consistently high prevalence of Acidobacteriota and Proteobacteria across varied environments, supporting the notion of their ecological significance. Prior studies have highlighted that the majority of ammonia-oxidizing bacteria belong to Proteobacteria; these play a crucial role in the nitrogen cycle, benefiting plant growth, yield, and fruit or seed quality [43]. Proteobacteria also dominate various soil ecosystems, including saline and semiarid soils [44]. The DF plantation is characterized by coastal saline conditions, and its soils have elevated salt content and pH compared to soils in the GM plantation. The presence of Proteobacteria in these soils may aid pecan trees in acclimating to coastal saline–alkali environments; however, further research is required to elucidate their specific mechanisms of action. Acidobacteriales members thrive in acidic soils [45]; this may account for their notable enrichment in GM plantation soils, which have lower pH levels. At the genus level, *Ca. Udaeobacter* exhibited significant enrichment and showed potential as a bacterial biomarker in the GM plantation, whereas *Bacillus* showed significant enrichment and showed potential as a bacterial biomarker in the DF plantation (Figure 4; Table S6). *Candidatus Udaeobacter*, a globally abundant verrucomicrobial clade, has demonstrated a preference for acidic soil environments [46]. *Bacillus*, the dominant genus of growth-promoting bacteria found in the plant rhizosphere, plays a crucial role in promoting plant growth and nutrient absorption, and in assisting plants in coping with saline–alkaline stress environments [47].

Ascomycota and Basidiomycota were found to be the dominant fungal phyla in both pecan plantations examined in this study, which is consistent with the findings of a previous study [41,48]. Interestingly, Ascomycetes were the most abundant taxon in DF plantation soil; however, the relative abundance of Ascomycetes decreased in GM plantation soil. In contrast, the relative abundance of Basidiomycetes increased greatly, and it became predominant in GM plantation soil (Figure 3; Table S2). The growth and distribution of basidiomycete fungi may be affected by adverse environmental conditions in coastal soil. Basidiomycetes generally tend to have lower salt tolerance than Ascomycetes [49]. Moreover, the majority of dominant fungi species have genomic potential for elevated competition, resource utilization, and stress tolerance, consequently leading to their increased dominance in different soil types [48,50]. Our findings revealed significant variation in fungal community composition between the pecan plantations at the genus level, unlike bacterial communities (Figure 3). Previous studies have shown that pecan trees can form symbiotic relationships with a wide range of soil fungi, particularly ectomycorrhizal fungi [41,51]. As pecan trees lack root hairs, ectomycorrhizal fungi fulfill a similar role in absorbing moisture and minerals [41]. In this study, ectomycorrhizal fungi constituted the majority of fungi at the genus level. The commensal association between trees and fungi underscores the importance of host specificity in shaping mycorrhizal communities [52]. Furthermore, previous investigations have shown that fungal communities are highly sensitive to habitat changes [53]. Host trees may select their symbiotic fungi according to the influence of varying growth environments [54]. This dependency may explain the observed sensitivity of fungal community composition at the genus level to environmental differences between pecan plantations. The ectomycorrhizal fungi associated with pecan trees commonly belong to the genera *Tuber*, *Scleroderma*, *Russula*, *Pisolithus*, *Tomentella*, *Inocybe*, and *Hebeloma* [51,55]. In this study, *Scleroderma* was identified as a fungal biomarker species in DF soils, with a notably higher abundance compared to GM soils (Figure 4; Table S6). This significant difference in abundance suggests that *Scleroderma* species may be integral to the core microbiome of pecan trees in alkaline environments, potentially playing a crucial role in their adaptation to these conditions. Ectomycorrhizal fungi demonstrate significant functional diversity, with various species exhibiting different responses to environmental conditions. In this study, *Clavulina* emerged as the most dominant genus in GM soils. Previous studies have indicated that *Clavulina* species are frequently found in areas with higher soil nutrient content [56,57], which aligns with our current findings (Figure 3; Table 1).

### 4.3. Relationships between Soil Chemical Properties and Microbial Community Structure

Soil physical and chemical properties are the main factors affecting soil microbial structure [6]. Significant differences in soil physicochemical properties were observed between the DF and GM plantations (Table 1), which subsequently influenced the proportions of soil microorganisms and their community structures. In this study, pH emerged as a critical regulator of both bacterial and fungal communities within pecan plantations, significantly impacting their composition (Figure 5; Table S3).

As a significant bacterial biomarker in DF pecan plantations, *Bacillus* was strongly positively correlated with soil pH (*p* < 0.05) and TSS (*p* < 0.01) (Figure 6; Table S6). Studies have shown that *Bacillus subtilis* can enhance rose growth and improve stress resistance under saline–alkaline conditions by regulating the root microbial community [58]. Additionally, certain *Bacillus* species exhibit a strong ability to dissolve inorganic phosphorus and are halophilic, displaying efficient phosphorus solubilization activity within a salinity range of 1–8% [59]. Thus, the presence of *Bacillus* bacteria may significantly aid pecan trees in adapting to coastal environments and coping with saline–alkali stress.

In this study, *Scleroderma* (*p* < 0.05), *Hebeloma* (*p* < 0.01), *Naucoria* (*p* < 0.01), and Mortierella (*p* < 0.01) were significantly positively correlated with soil pH (Figure 6). Many nursery ectomycorrhizal fungi, such as *Thelephora* spp., *Cenococcum* spp., *Hebeloma* spp., and *Scleroderma* spp., have been shown to thrive at pH levels close to 8 [60]. The average pH of DF soils was approximately 8.04, whereas for GM soils it was ca. 5.60 (Table 1). Early studies reported that *Laccaria laccata* exhibited lower efficacy in promoting growth compared to *Pisolithus* spp. across a range of pH levels (4.6–6.6), whereas *Scleroderma cepa* showed no significant effect across the same pH range relative to uninoculated control treatments [61]. Although that study did not specifically address the performance of *S*. *cepa* at higher pH levels, this finding suggests a broader soil pH adaptability range for *S*. *cepa*. A previous study reported that *Scleroderma* species are the most frequently detected basidiomycetes in pecan orchards, with their frequency and abundance increasing at higher soil pH levels [51]. *Naucoria* is considered to possess characteristics of ectomycorrhizal fungi and is commonly found in saline forest sites [62]. In addition to being ectomycorrhizal fungi, *Naucoria* fungi are associated with plant roots in a mutually beneficial relationship, aiding nutrient uptake and enhancing plant growth. This relationship may be particularly advantageous for plants growing in saline forest sites, where nutrient availability is often limited. Certain strains of *Mortierella* are part of the plant growth-promoting fungus (PGPF) group and are predominant within fungal communities in saline–alkaline soil [63]. These fungi can solubilize phosphate and enhance soil quality in coastal mudflat areas [64,65]. The presence of *Mortierella* species as PGPFs in saline–alkaline soil suggests their potential role in improving soil quality and supporting plant growth in challenging environments.

Despite artificial amelioration efforts to reduce salt content in the soils of the coastal DF plantation, salt levels remained higher in the saline environment of DF plantation soils compared to GM plantation soils (Table 1). Although soil TSS did not have a significant effect on the diversity of soil bacterial or fungal communities, the alpha diversity levels of both bacteria and fungi were positively correlated with TSS. Interestingly, we found that *Scleroderma* species were significantly positively correlated with TSS (*p* < 0.001) (Figure 6). Salinity tolerance traits have been investigated mainly within Basidiomycota, and the results have revealed that these traits are not consistently associated with specific clades. Rather, they are commonly observed within individuals of the same species or even genus [49]. Previous studies have demonstrated that the salt tolerance of seagrape (*Coccoloba uvifera*) is significantly improved by the presence of *Scleroderma bermudense* under both varying salt stress conditions and controlled conditions [66]. Under non-saline conditions, pecan seedlings exhibited enhanced physiological responses and increased biomass accumulation when inoculated with a *Scleroderma* species. Under saline conditions, inoculation with *Scleroderma* sp. was found to significantly increase the potassium ion (K^+^) content of pecan seedling leaves, particularly as the salt concentration increased from 20 to 35 mM NaCl; higher salt concentrations were associated with higher leaf K^+^ content in pecan seedlings [67]. This finding suggests that adequate K^+^ nutrition facilitated by ectomycorrhizal symbiosis plays a crucial role in improving plant adaptation to salt stress [49,68].

Ectomycorrhizal fungi exhibit significant functional diversity, with responses to environmental conditions varying among species. For example, a recent study found that *Russula* species were exclusively found at non-saline control sites, whereas the number of ectomycorrhizal tips of *Cortinarius* species decreased with increasing salinity; overall, the *Russula* and *Cortinarius* species demonstrated lower salinity tolerance compared to the *Thelephora* and *Tomentella* species [62]. Besides soil pH and TSS, other environmental factors, such as mineral nutrient levels, also influence the composition and abundance of soil microbial communities [69]. In this study, *Clavulina* and *Russula* were the dominant fungal genera in GM soils, which had higher mineral nutrient levels (AN, AP, AK) than DF soils. Genus *Russula* includes both the nitrophobic and nitrophilic species [70]. Additionally, *Clavulina* species have been observed in areas with higher soil nutrient content [56]. Thus, differences in soil pH, mineral nutrient levels, and salinity contribute to the distinct soil microbial community structures between coastal and inland pecan plantations.

Soil salinity and alkalinity often coincide, and the levels of certain mineral nutrients in the soil are typically associated with soil pH. Consequently, it is challenging to isolate the impact of each individual factor. Our analysis suggests that pH is the most crucial factor in soil microbial community structure in pecan plantations. Additionally, positive correlations were detected between certain bacterial and fungal genera and both pH and TSS, indicating their potential role in helping pecan trees adapt to coastal environments and mitigate saline–alkali stress. However, future studies should experimentally examine the individual and combined effects of soil pH, TSS, and mineral nutrients on soil microbial communities, particularly ectomycorrhizal fungi.

### 4.4. Soil Microbial Network Structures and Functions in Pecan Plantations

Regardless of the plantation type, 16S–ITS link patterns played a prominent role in shaping the microbial networks examined in this study. Overall, the prevalence of positive links exceeded that of negative links, indicating the dominant influence of co-occurrence patterns in the microbial network (Figure 7; Table S4). Furthermore, GM plantation soils exhibited simpler network topologies in terms of bacterial and fungal communities compared to DF plantation soils (Table S5). Although the relatively high salinity and pH in coastal soils filtered out some soil bacteria and fungi, they may have increased the abundance of halophilic microorganisms. Thus, the adverse coastal growth environment likely resulted in a more complex soil bacterial network [13]. Previous studies have also shown that high-salinity soils host diverse salt-tolerant microbial communities, with a symbiotic network analysis revealing stronger dependencies among species associated with severely salinized soils [71].

Our analysis revealed that bacterial communities had the highest functional abundances in carbohydrate metabolism, lipid metabolism, and glycan biosynthesis and metabolism (Figure 8a). This is likely because the GM plantation soils had higher contents of SOM and nutrients such as AN, AP, and AK compared to DF plantation soils, which gave soil bacteria in the GM plantation a greater capacity for carbohydrate metabolism. The carbon source for soil bacterial activity is primarily derived from litter and root exudates [72]. Previous studies have identified two bacterial genera, *Ca. Solibacter* (Verrucomicrobia) and *HSB_OF53-F07* (Chloroflexi), as being involved in organic matter decomposition and carbon source utilization [73,74,75].

A FunGuild analysis of fungal functions revealed that ectomycorrhizal fungi exhibited the highest relative abundance, with a greater presence in the DF plantation compared to the GM plantation (Figure 8). These fungi, which are associated with plant roots in a mutually beneficial relationship, aid nutrient uptake and enhance plant growth [51]. This mutualism may be particularly advantageous for plants growing in saline forests where nutrient availability is often limited.

Investigating whether the ability of pecan trees to withstand saline-alkaline conditions is influenced by their interactions with ectomycorrhizal fungi, such as *Scleroderma* spp., and other soil microorganisms, as well as the extent of this influence, would be worthwhile. Exploring this relationship could provide valuable insights into optimizing plant–fungus interactions and enhancing plant adaptability in saline–alkaline environments. From an ecological perspective, utilizing appropriate ectomycorrhizal fungi in conjunction with corresponding functional bacteria is crucial for improving the survival rate of pecan seedlings in coastal saline soils. This approach would promote the development of ecologically sustainable agroforestry industries and contribute to the amelioration of coastal marshland ecosystems. It is important to note that this study was conducted on only two pecan plantations and may not be representative of other sites. Therefore, further research is needed to confirm whether these results are consistent across additional locations.

## 5. Conclusions

This study revealed that soils sampled from coastal pecan plantations exhibited higher microbial diversity than those sampled from inland pecan plantations, and this was significantly influenced by pH and other edaphic factors. Notably, the abundances of bacterial *Bacillus* and fungal *Scleroderma* were distinct in the soils of pecan trees grown in coastal fields, identifying these groups as genus-level biomarkers for coastal environments. A functional analysis indicated greater potential for carbohydrate metabolism in GM plantation bacteria and a higher abundance of ectomycorrhizal fungi in DF soil. Positive correlations between certain bacterial and fungal genera, and pH and TSS, suggested their roles in pecan adaptation to coastal environments and saline–alkali stress mitigation.

Although the commonality of these results in other pecan plantations needs further confirmation, our findings enhance the understanding of soil microbiomes in coastal pecan plantations, highlighting the importance of utilizing specific microbial communities to promote ecologically sustainable agroforestry practices and contributing to the management of coastal marshland ecosystems.

## Figures and Tables

**Table 1:** Soil physicochemical properties in different pecan plantations.

Soil Physicochemical Properties	DF	GM
TN (g/kg)	0.42 ± 0.02 b	0.65 ± 0.02 a
TP (g/kg)	0.66 ± 0.03 a	0.48 ± 0.01 b
TK (g/kg)	13.58 ± 0.45 a	9.49 ± 0.56 b
AN (mg/kg)	36.27 ± 1.02 b	69.74 ± 2.35 a
AP (mg/kg)	38.83 ± 0.15 b	62.06 ± 4.95 a
AK (mg/kg)	93.58 ± 0.05 b	117.26 ± 5.84 a
SOM (g/kg)	8.44 ± 0.33 b	12.94 ± 0.14 a
TSS (g/kg)	0.86 ± 0.24 a	0.37 ± 0.03 b
pH	8.04 ± 0.02 a	5.60 ± 0.41 b
EC (μs/cm)	220.00 ± 3.61 a	121.87 ± 15.55 b

Note: TN, total nitrogen; TP, total phosphorus; TK, total potassium; AN, available nitrogen; AP, available phosphorus; AK, available potassium; SOM, soil organic matter; TSS, total soluble salts; EC, electrical conductivity. Different letters indicate significant differences among different plantations (*p* < 0.05 after Benjamini–Hochberg adjustment; paired *t*-test). The abbreviations DF and GM correspond to the Dafeng forest farm and the Guomei pecan plantation, respectively.

## Data Availability

The raw sequencing reads are available in the NCBI Sequence Read Archive (SRA) under the accession numbers PRJNA1117251 for bacteria and PRJNA1117621 for fungi.

## Supplementary Materials

The following supporting information can be downloaded at: https://www.mdpi.com/article/10.3390/microorganisms12071313/s1, Table S1: Sequencing information statistics for each sample; Table S2: Statistical analysis of top 10 bacterial and fungal phyla or genera in pecan plantations; Table S3: Identification of chemical properties correlated with bacterial and fungal communities via envfit analysis; Table S4: Nodes and links of co-occurrence networks in different pecan plantations; Table S5: Topological properties of soil microbial (bacteria and fungi) networks in different pecan plantations; Table S6: Keystone taxa in different pecan plantations.
