# Supplementary material for: A Comparative Analysis of Bacterial and Fungal Communities in Coastal and Inland Pecan Plantations
Source: Microorganisms. 2024 Jun 27;12(7):1313. doi: 10.3390/microorganisms12071313 (PMC11279223; doi:10.3390/microorganisms12071313)
Supplement: Supplementary file 1 [file microorganisms-12-01313-s001.zip › microorganisms-3072322-supplementary/Table S1 Sequencing information statistics for each sample.pdf]

Table S1 Sequencing information statistics for each sample

	Sample ID	Tags number	Total length	Max length	Min length	N50	N90	ASVs
Bacteria	DF1	85121	35611555	441	212	440	433	16446
	DF2	86545	36177618	441	207	434	432	16037
	DF3	88113	36923138	441	212	434	432	16607
	GM1	92293	37947363	441	212	432	432	13688
	GM2	83797	34851823	441	227	439	432	13883
	GM3	88813	36849619	441	218	433	432	13304
	Total	524682						89965
Fungi	DF1	109431	37212541	440	175	409	405	736
	DF2	111282	39257175	439	194	407	405	705
	DF3	116010	41046490	434	210	422	414	612
	GM1	101456	36510387	416	194	402	402	575
	GM2	123616	41541075	433	271	402	402	425
	GM3	110574	38965540	409	247	402	402	615
	Total	672369						3668

Tags number: the number of effective sequences of clean tags after filtering the chimera; Total length: total length of tags; Max length: the length of the longest tag; Min length: the length of the shortest tag; N50: the N50 length of the sample tags sequence; N90: the N90 length of the sample tags sequence; ASVs: The final number of amplicon sequence variants (ASVs) obtained.
