# Supplementary material for: A Comparative Analysis of Bacterial and Fungal Communities in Coastal and Inland Pecan Plantations
Source: Microorganisms. 2024 Jun 27;12(7):1313. doi: 10.3390/microorganisms12071313 (PMC11279223; doi:10.3390/microorganisms12071313)
Supplement: Supplementary file 1 [file microorganisms-12-01313-s001.zip › microorganisms-3072322-supplementary/Table S2 Statistical analysis of top 10 bacterial and fungal phyla or genera in pecan plantations.pdf]

**Table S2** Statistical analysis of top 10 bacterial and fungal phyla or genera in pecan plantations

| | Phylum | | | Genus | | |
|---|---|---|---|---|---|---|
| | | DF | GM | | DF | GM |
| **Bacteria** | Acidobacteriota | b | a** | *RB41* | a | ab |
| | Proteobacteria | a* | b | *Candidatus_Udaeobacter* | de | a** |
| | Chloroflexi | cd | bc | *Nitrospira* | b** | c |
| | Verrucomicrobiota | ef | bc*** | *Bacillus* | bc* | c |
| | Bacteroidota | c | c | *ADurbBin063-1* | e | bc*** |
| | Planctomycetota | de** | cd | *UTCFX1* | cd* | c |
| | Actinobacteriota | f | cd | *HSB_OF53-F07* | e | bc* |
| | Patescibacteria | de*** | cd | *Bryobacter* | e | bc** |
| | Gemmatimonadota | f | cd | *Candidatus_Solibacter* | e | bc*** |
| | Firmicutes | f* | d | *Sphingomonas* | de | c |
| | Other | – | – | Other | – | – |
| **Fungi** | | DF | GM | | DF | GM |
| | Basidiomycota | a | a* | *Scleroderma* | a* | b |
| | Ascomycota | b* | b | *Clavulina* | b | a** |
| | Mortierellomycota | c** | c | *Sphaerosporella* | ab | b |
| | Monoblepharomycota | c* | c | *Russula* | b | b* |
| | Rozellomycota | c | c | *Astraeus* | b | b |
| | Chytridiomycota | c** | c | *Pisolithus* | b | b |
| | Mucoromycota | c | c | *Hebeloma* | b*** | b |
| | Glomeromycota | c | c | *Naucoria* | b*** | b |
| | Aphelidiomycota | c | c | *Inocybe* | b | b* |
| | Kickxellomycota | c | c | *Sebacina* | b | b |
| | Other | – | – | *Other* | – | – |

Statistical analysis was conducted to determine the relative abundance of the top 10 bacterial and fungal phyla or genera in various pecan plantations. The differences in relative abundance of different microorganisms（bacteria and fungi）at the phylum or genus level within the same pecan plantation are represented by different lowercase letters ($P<0.05$). *, **, *** indicate that the difference between Dafeng forest farm (DF) and Guomei pecan plantation (GM) under the same phylum or genus is significant at $P<0.05$, 0.01 and 0.001 level, respectively.
