# Supplementary material for: A Comparative Analysis of Bacterial and Fungal Communities in Coastal and Inland Pecan Plantations
Source: Microorganisms. 2024 Jun 27;12(7):1313. doi: 10.3390/microorganisms12071313 (PMC11279223; doi:10.3390/microorganisms12071313)
Supplement: Supplementary file 1 [file microorganisms-12-01313-s001.zip › microorganisms-3072322-supplementary/Table S3 Identification of chemical properties correlated with bacterial and fungal communities via envfit a.pdf]

Table S3 Identification of chemical properties correlated with bacterial and fungal communities via envfit analysis

	Bacteria				Fungi			
	CCA1	CCA2	r2	pvals	CCA1	CCA2	r2	pvals
TN	0.9997	0.0242	0.9756	0.0931	0.9998	-0.0205	0.9803	**0.0458**
TP	-0.9964	-0.0852	0.9702	**0.0375**	-0.9987	0.0510	0.9312	0.1000
TK	-0.9978	-0.0662	0.9597	0.0847	-0.9997	-0.0262	0.9735	**0.0389**
AN	0.9971	-0.0758	0.9960	**0.0194**	0.9916	-0.1294	0.9885	0.0597
AP	0.9640	0.2659	0.9815	**0.0125**	0.9991	0.0422	0.9447	0.0667
SOM	0.9998	0.0182	0.9932	0.0583	0.9978	-0.0665	0.9847	0.0694
AK	0.9973	-0.0741	0.9098	0.0917	0.9559	-0.2937	0.9438	**0.0375**
TSS	-0.9982	-0.0595	0.8072	0.0917	-0.9947	0.1024	0.7829	0.1111
EC	-0.9870	-0.1607	0.9744	**0.0389**	-0.9989	0.0465	0.9505	0.0861
pH	-0.9911	0.1333	0.9724	**0.0403**	-0.9614	0.2750	0.9856	**0.0097**

An envfit analysis utilizing the envfit function identifies chemical properties that are correlated with bacterial and fungal communities ($p < 0.05$). TN, total nitrogen; TP, total phosphorus; TK, total potassium; AN, available nitrogen; AP, available phosphorus; AK, available potassium; SOM, soil organic matter; TSS, total soluble salts; EC, electrical conductivity.
