# Supplementary material for: A Comparative Analysis of Bacterial and Fungal Communities in Coastal and Inland Pecan Plantations
Source: Microorganisms. 2024 Jun 27;12(7):1313. doi: 10.3390/microorganisms12071313 (PMC11279223; doi:10.3390/microorganisms12071313)
Supplement: Supplementary file 1 [file microorganisms-12-01313-s001.zip › microorganisms-3072322-supplementary/Table S4 Nodes and links of co-occurrence networks in different pecan plantations.pdf]

Table S4 Nodes and links of co-occurrence networks in different pecan plantations

	DF	GM
Bacterial nodes (proportion)	99 (50.5%)	99 (49.7%)
Fungal nodes (proportion)	97 (49.5%)	100 (50.3%)
Total nodes	196	199
16S-16S links (proportion)	257 (24.3%)	245 (22.6%)
ITS-ITS links (proportion)	287 (27.1%)	283 (26.0%)
16S-ITS links (proportion)	514 (48.6%)	558 (51.4%)
Total links	1058	1086
16S-16S positive links (proportion)	148 (27.0%)	142 (20.2%)
ITS-ITS positive links (proportion)	155 (28.3%)	236 (33.6%)
16S-ITS positive links (proportion)	245 (44.7%)	324 (46.2%)
Total positive links	548 (51.8%)	702 (64.6%)
16S-16S negative links	109 (21.4%)	103 (26.8%)
ITS-ITS negative links	132 (25.9%)	47 (12.2%)
16S-ITS negative links	269 (52.7%)	234 (61.0%)
Total negative links	510 (48.2%)	384 (35.4%)

The co-occurrence networks were constructed based on the bacterial(16S) and fungal (ITS) Classes, with a significance level of $p < 0.05$ and a Pearson coefficient size > 0.6.
