# Supplementary material for: A Comparative Analysis of Bacterial and Fungal Communities in Coastal and Inland Pecan Plantations
Source: Microorganisms. 2024 Jun 27;12(7):1313. doi: 10.3390/microorganisms12071313 (PMC11279223; doi:10.3390/microorganisms12071313)
Supplement: Supplementary file 1 [file microorganisms-12-01313-s001.zip › microorganisms-3072322-supplementary/Table S5 Topological properties of soil microbial (bacteria and fungi) networks in different pecan plantatio.pdf]

Table S5 Topological properties of soil microbial (bacteria and fungi) networks in different pecan plantations

	DF	GM
Number of nodes	196	199
Number of edges	1058	1086
Positive correlation	0.52	0.65
Negative correlation	0.48	0.35
Graph density	0.053	0.055
Mean degree	10.58	10.86
Average path length	9.28	8.87
Connected components	4	3
Transitivity	0.75	0.76
Average clustering coefficient	0.77	0.76
Edge connectivity	0	0
Centralize betweenness	0.18	0.20
Centralize degree	0.037	0.036
Diameter	30	28
Edge connectivity	0	0
Reciprocity of graphs	1	1
Community structure	8	8
Modularity	0.77	0.79

The network displays co-occurring bacteria and fungi, which have been identified through rigorous correlation analysis. For this analysis, the top 500 Amplicon Sequence Variants (ASVs) were chosen. The Pearson's correlation coefficient value between the ASVs is greater than 0.9, with a significance level of $p < 0.05$.
