# Supplementary material for: A Comparative Analysis of Bacterial and Fungal Communities in Coastal and Inland Pecan Plantations
Source: Microorganisms. 2024 Jun 27;12(7):1313. doi: 10.3390/microorganisms12071313 (PMC11279223; doi:10.3390/microorganisms12071313)
Supplement: Supplementary file 1 [file microorganisms-12-01313-s001.zip › microorganisms-3072322-supplementary/Table S6 Keystone taxa in different pecan plantations.pdf]

Table S6 Keystone taxa in different pecan plantations

Plantations	Microbial taxa	ASV	Degree	Phylum	Genus
DF	Bacteria	16S-ASV000024	17	p__Acidobacteriota	g__RB41
		16S-ASV000013	17	p__Firmicutes	g__Bacillus
		16S-ASV000058	17	p__Chloroflexi	g__UTCFX1
		16S-ASV000305	17	p__Acidobacteriota	f__ Vicinamibacteraceae_g__ unclassified
		16S-ASV000005	16	p__Bacteroidota	g__Chryseolinea
	Fungi	ITS-ASV000070	17	p__Basidiomycota	g__Scleroderma
		ITS-ASV000160	17	p__Ascomycota	g__Striaticonidium
		ITS-ASV000251	17	p__Ascomycota	g__Nigrograna
		ITS-ASV000107	16	p__Basidiomycota	g__Paulisebacina
		ITS-ASV000110	16	p__Basidiomycota	g__Hebeloma
GM	Bacteria	16S-ASV000088	17	p__Acidobacteriota	g__RB41
		16S-ASV000125	17	p__Acidobacteriota	g__Bryobacter
		16S-ASV000081	16	p__Verrucomicrobiota	g__Candidatus_Udaeobacter
		16S-ASV000152	16	p__Verrucomicrobiota	g__Candidatus_Xiphinematobacter
		16S-ASV000066	16	p__ Acidobacteriota	f__Acidobacteriaceae_Subgroup_1 _g__ unclassified
	Fungi	ITS-ASV000021	18	p__Basidiomycota	g__Sebacina
		ITS-ASV000117	18	p__Basidiomycota	g__Mycena
		ITS-ASV000278	18	p__Basidiomycota	g__Sebacina
		ITS-ASV000036	17	p__Basidiomycota	o__Agaricales_g__ unclassified
		ITS-ASV000270	17	p__Mortierellomycota	g__Mortierella

The table displays the top 5 node degrees, ranked from highest to lowest, for bacterial and fungal ASVs in the correlation network analysis. In this table, p, o, f and are used to denote phylum, order, family and genus, respectively. In cases where there is no annotation at the genus level, the name of the higher level to which it is annotated is presented.
